# Influence of Solution Properties and Process Parameters on the Formation and Morphology of YSZ and NiO Ceramic Nanofibers by Electrospinning

**DOI:** 10.3390/nano7010016

**Published:** 2017-01-13

**Authors:** Gerard Cadafalch Gazquez, Vera Smulders, Sjoerd A. Veldhuis, Paul Wieringa, Lorenzo Moroni, Bernard A. Boukamp, Johan E. ten Elshof

**Affiliations:** 1 MESA+ Institute for Nanotechnology, University of Twente, P.O. Box 217, 7500 AE Enschede, The Netherlands; gerard.cadafalch@eurekite.com (G.C.G.); v.smulders@utwente.nl (V.S.); sveldhuis@ntu.edu.sg (S.A.V.); b.a.boukamp@utwente.nl (B.A.B.); 2 Department of Complex Tissue Regeneration, MERLN Institute for Technology Inspired Regenerative Medicine, University of Maastricht, 6200 MD, Maastricht, The Netherlands; p.wieringa@maastrichtuniversity.nl (P.W.); l.moroni@maastrichtuniversity.nl (L.M.)

**Keywords:** yttria-stabilized zirconia, YSZ, nickel oxide, NiO, nanofiber, electrospinning, microstructure, spinning process, alignment

## Abstract

The fabrication process of ceramic yttria-stabilized zirconia (YSZ) and nickel oxide nanofibers by electrospinning is reported. The preparation of hollow YSZ nanofibers and aligned nanofiber arrays is also demonstrated. The influence of the process parameters of the electrospinning process, the physicochemical properties of the spinning solutions, and the thermal treatment procedure on spinnability and final microstructure of the ceramic fibers was determined. The fiber diameter can be varied from hundreds of nanometers to more than a micrometer by controlling the solution properties of the electrospinning process, while the grain size and surface roughness of the resulting fibers are mainly controlled via the final thermal annealing process. Although most observed phenomena are in qualitative agreement with previous studies on the electrospinning of polymeric nanofibers, one of the main differences is the high ionic strength of ceramic precursor solutions, which may hamper the spinnability. A strategy to control the effective ionic strength of precursor solutions is also presented.

## 1. Introduction

Ceramic nanofibers have gained considerable interest during the last decade. Their large surface area and aspect ratio provide superior properties for a wide range of applications, such as catalysis, energy, biomedicine, sensing, or electronics [1,2,3]. Among the different nanofiber preparation methods, electrospinning appears to be the most feasible one due to the relatively uncomplicated equipment, flexibility in composition, control over fiber characteristics, and higher production rates than are possible with other methods [1,2,4,5,6].

**Figure 1:**
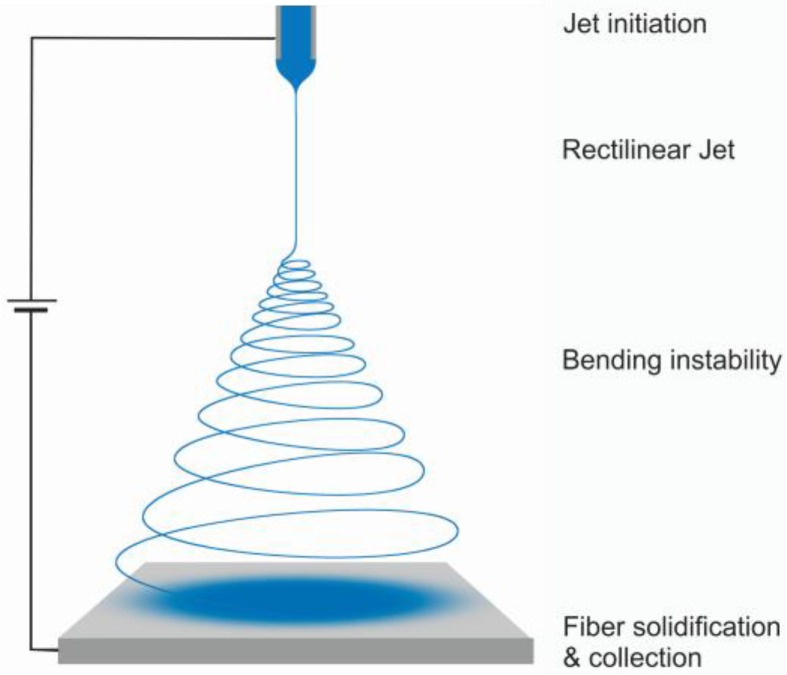
Schematic representation of the electrospinning process.

A typical electrospinning setup essentially consists of a spinneret connected to a counter electrode collector via a high voltage supply (Figure 1) [7,8]. A viscous material solution is pumped into a nozzle, the subsequent applied electric field promotes the fluid to overcome the surface tension of the droplet at the tip of the spinneret, and the droplet forms a Taylor cone. The viscosity of the solution prevents the formation of separate droplets, allowing a single fiber to be drawn from the solution [7,9]. Then, the jet shrinks in diameter to form micro and nanofibers that dry and get collected on the counter electrode [7,8,10,11]. The electrospinning process can be divided into four stages [8,10]: jet initiation, rectilinear jet, bending instability, and fiber solidification and collection (Figure 1). During the jet initiation stage, repulsive Coulomb interactions dominate the Taylor cone formation [8,12]. In the rectilinear jet stage, the viscoelastic forces and surface tension compensate for any perturbations of the jet [11]. However, at a certain point the viscosity can no longer stabilize the perturbations and whipping occurs, known as the bending instability stage. At this point the jet starts whipping in a circular motion, with each consecutive circle larger than the previous one. It is considered that fiber thinning mostly happens in this stage [11]. Finally, the whipping jet reaches the collector plate and deposits the fibers on its surface. Upon touching the electrode, trapped electrostatic charges start being released to the ground [8,13,14].

Ceramic nanofibers can be formed when a suitable precursor is introduced in the solution. Initially, organic-inorganic hybrid fibers are spun. Then, a thermal treatment is applied to the green fibers to burn out the organic compounds, and form dense crystalline ceramic fibers [1,2]. However, achieving good control over the resulting microstructure remains a challenge and the process is not fully understood [8,15]. The reproducibility of the process should also be further improved in order to provide an economically feasible route to production [1].

The fabrication of ceramic nanofibers by electrospinning has been reviewed elsewhere [1,2,16,17]. These papers describe the fabrication of ceramic nanofibers of diverse compositions, fabrication methods and applications. Nonetheless, all theoretical and parametric studies of electrospinning and setup assemblies available so far are based on the formation of polymeric fibers [7,8].

Here we report a systematic parametric study on the formation of ceramic nanofibers by the electrospinning process. Yttria-stabilized zirconia (YSZ) and nickel oxide (NiO) were taken as model compounds, and both dense and hollow YSZ fibers are reported. Yttria-stabilized zirconia has a wide range of applications: it is thermally and chemically very stable, a good ionic conductor at high temperatures, and used in thermal barrier coatings, while NiO is a semiconductor and has been proposed as photoanode. The most prominent difference between the YSZ and NiO precursor solutions is the ionic strength of the solution, which strongly affects the spinnability of the fibers. The physicochemical properties of the precursor solutions and the main process parameters of both types of solution were varied systematically to determine their influence on spinnability, and on the microstructure and morphology of the resulting fibers. The experimental data are compared with literature data to interpret the results. We confirmed the applicability of theoretical studies on electrospinning of polymers on the formation of arrays of metal oxide nanofibers.

## 2. Results and Discussion

### 2.1. Solution Properties 

**Figure 2:**
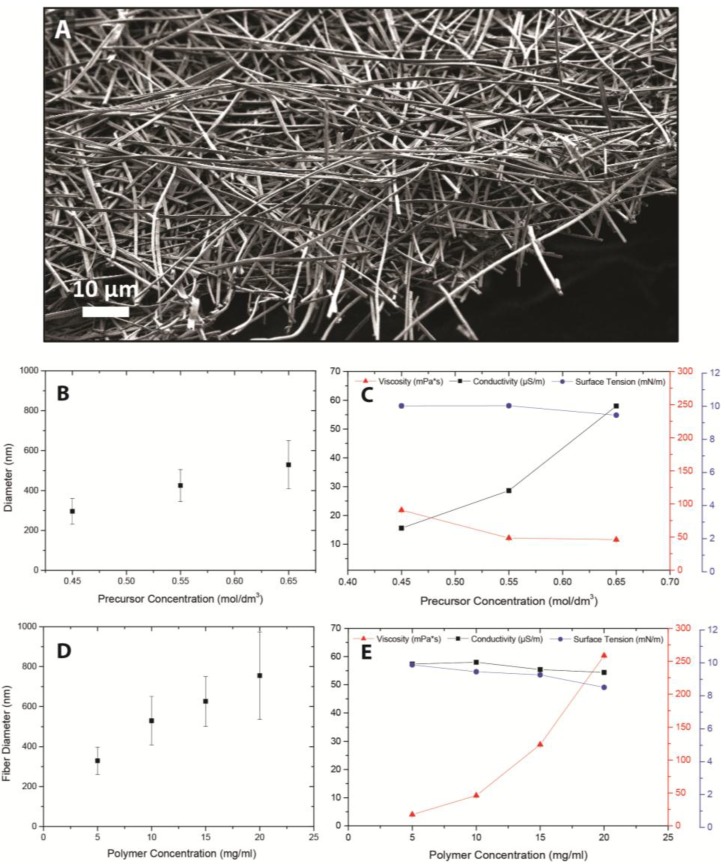
(**A**) Scanning electron microscopy (SEM) picture of three percent yttria partially-stabilized zirconia (3YSZ) fibers. Precursor concentration 0.65 M, polymer concentration 10 mg/mL polyvinylpyrrolidone (PVP). Influence of precursor concentration on (**B**) fiber diameter (nm) and (**C**) solution conductivity (μS/m; black squares), static viscosity (mPa·s; red triangles) and surface tension (mN/m; blue circles). Polymer concentration 10 mg/mL. Influence of polymer concentration on (**D**) fiber diameter (nm) and (**E**) solution conductivity (μS/m; black squares), static viscosity (mPa·s; red triangles) and surface tension (mN/m; blue circles). Precursor concentration 0.65 M.

The electrospinning process typically yielded fibers as shown in Figure 2A. The fibers shown were highly disordered and their average diameter was 530 ± 121 nm as determined from statistical analysis of the scanning electron microscopy (SEM) pictures. The physical properties of the solution played a crucial role in the electrospinning process [1,18,19]. The main solution properties affecting the electrospinning process are viscosity η, conductivity σ, surface tension γ, and solvent volatility [8,10,11,19]. We measured these physical properties experimentally. The volatility can be extrapolated from the solvent’s boiling point [19,20,21,22]. We investigated the influence of both the polymer and precursor concentration on the solution properties and the resulting fibers. The results of these experiments in which the polymer concentration was kept constant while the precursor concentration was varied between 0.45 and 0.65 M are shown in Figure 2B,C.

The viscosity and surface tension did not change significantly with precursor concentration. A linear correlation between the precursor concentration and fiber diameter after drying and thermal treatment is found. The fiber diameter varied from 300 ± 44 nm at low precursor concentration to 530 ± 120 nm at high concentration. The fibers formed from the solutions with lowest precursor concentration included beads of a few micrometers in size. The conductivity increased with precursor concentration. This can be attributed to the higher concentration of free ions and other species susceptible to ionization.

The results indicate that the fiber diameter is primarily dependent on the equivalent solids content after thermal annealing. Although high conductivity might result in thinner polymeric or green fibers [19], the influence of solution conductivity on fiber diameter after annealing was found to be much less prominent than the solids content. Previous studies on ceramic fibers with different composition showed a similar trend [1,15].

Beaded fibers have been attributed to low viscosities [18,19] in polymer electrospinning experiments. However, in our experiments we did not observe a relationship between viscosity and the occurrence of beaded fibers. A stable jet results from a balance between viscoelasticity, surface tension and electrostatic forces, besides the pressure drop due to the pump [12,23]. The electrostatic force is mainly dictated by the conductivity of the solution in a given electric field [8]. The beaded fibers resulted from solutions with low conductivity, which had a similar viscosity and surface tension as solutions that produced fibers without beads. These findings suggest that the force balance between viscosity, surface tension and conductivity was shifted so that the jet could no longer maintain a stable fiber shape, which finally resulted in the formation of beads. Indeed, previous studies have shown that formation of beaded polymeric fibers can be avoided by the addition of soluble salts to a solution in order to increase its conductivity [19,22,24].

The influence of polymer concentration on solution properties and the resulting fibers is presented in Figure 2D,E. We observed an increase of fiber diameter with PVP concentration from 330 ± 68 nm to 754 ± 220 nm. The viscosity of the solution increased considerably with increasing polymer concentration, while it did not have a significant influence on the conductivity or the surface tension. The sample with high concentration of 20 mg/mL PVP was too viscous given the low surface tension, and this resulted in a slightly unstable jet producing a wide range of fiber diameters. This may have been the result of a force balance shift that hampered the jet stability [12,23].

**Figure 3:**
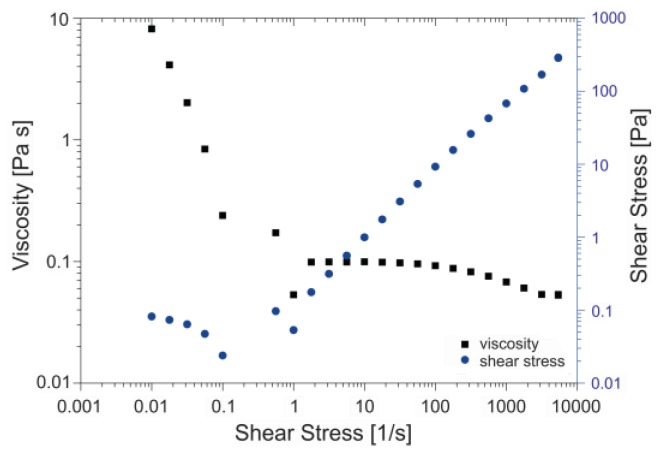
Dynamic viscosity data of the standard solution. Precursor concentration, 0.65 M; PVP concentration, 10 mg/mL.

We found that the influence of polymer concentration on viscosity is the main parameter that determines the fiber diameter in these experiments. A high viscosity stabilizes the jet and hinders the whipping phenomenon, which is considered to be the main cause of thinning and fiber formation [10,11]. The dynamic viscosity of the standard solution in Figure 3 shows shear thinning behavior at high shear rates. This means that while the jet is whipping, i.e., high shear, the dynamic viscosity of the solution decreases, which may allow the electrostatic instabilities to exert a larger influence. Nevertheless, the rapid viscosity increase is also determined by the high evaporation rate in the whipping stage.

Typically, solutions for electrospinning of ceramics contain a metal alkoxide precursor that condenses and forms a polymer-like network [1,25]. Such solutions can be considered as weak electrolytes, similar to polymeric solutions with low conductivity [8]. In contrast, complete dissociation of salts, such as metal nitrates, which are also common ceramic precursors, leads to strong electrolytes in aqueous solution. Such strong electrolytes cannot be easily electrospun due to their large conductivity. The model system we investigated was NiO made from nickel nitrate solutions.

**Figure 4:**
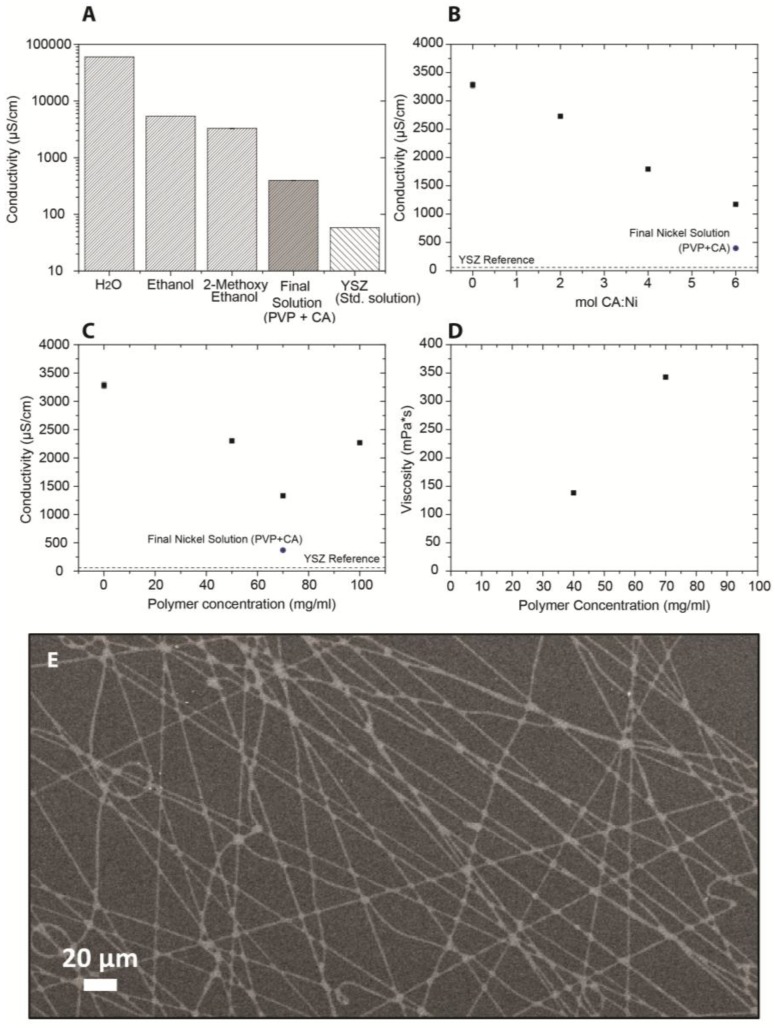
(**A**) Conductivity of a 0.21 M Ni(NO_3_)_2_ solution in various solvents; (**B**) conductivity of a 0.21 M Ni(NO_3_)_2_ solution in 2-ME at different molar ratios citric acid (CA):Ni; (**C**) conductivity of a 0.21 M Ni(NO_3_)_2_ solution in 2-ME with different polymer concentrations; (**D**) viscosity of a 0.21 M Ni(NO_3_)_2_ solution in 2-ME with different polymer concentrations. The conductivity of the final 0.21 M Ni(NO_3_)_2_ solution and the standard 3YSZ are also plotted as reference; and (**E**) electrospun NiO microfibers from a 0.21 M Ni(NO_3_)_2_ solution in 2-ME with 70 mg/mL of PVP at a molar ratio CA:Ni of 6:1.

The conductivity of a 0.21 M solution of nickel nitrate in milliQ water was 60.1 × 10^4^ ± 30 µS/cm, which is three orders of magnitude larger than that of the three percent yttria partially-stabilized zirconia (3YSZ) solution (58 ± 0.07 µS/cm), as discussed above. To reduce the solution’s conductivity, nickel nitrate was dissolved in other solvents at the same concentration. In ethanol and 2-ME, the conductivity reduced to 5.41 × 10^3^ ± 5 µS/cm and 3.28 × 10^3^ ± 58 µS/cm, respectively (Figure 4A). The lower conductivity results from the lower polarity of the latter solvents compared to water, which reduces the degree of dissociation of metal salts, and the smaller acid dissociation constant of the respective solvents. Moreover, 2-ME is widely used as a complexing agent for the stabilization of metal ions and metal alkoxide precursors [26,27]. Complexing agents can reduce the concentration of unbound ions in the solution and, consequently, lower the conductivity. Here, citric acid (CA) was used as additional complexing agent. Figure 4B shows a linear decrease of conductivity with increasing molar ratio of CA to Ni. The conductivity in 2-ME decreased from 3.28 × 10^3^ ± 58 µS/cm without CA to 1.17 × 10^3^ ± 3 µS/cm at a CA-to-Ni molar ratio of 6.

We also investigated the influence of the PVP concentration on the conductivity and viscosity of the nickel nitrate solution in 2-ME (without additional complexing agents), see Figure 4C. Increasing the PVP concentration resulted in a decrease of conductivity to 1.13 × 10^3^ ± 4 µS/cm at a PVP concentration of 70 mg/mL. However, upon further increase of the polymer content to 100 mg/mL, the conductivity increased to 2.27 × 10^3^ ± 2 µS/cm. This inversion of the trend can be understood by considering that PVP acts as a complexing agent for ions at low concentrations [28]. Hence, a decreasing conductivity is expected with increasing polymer content due to increasing degree of complexation. However, most Ni ions are part of some complex with PVP beyond a certain concentration threshold, and since the polymer itself is also ionically charged, it contributes to the total conductivity when it is in the solution in unbound form. Any further addition of PVP will thus result in a conductivity increase [29]. The viscosity increased with PVP concentration (Figure 4D), similar to the 3YSZ solution.

Nickel oxide fibers were successfully spun using a solution of 0.21 M nickel nitrate in 40:60 isopropanol:2-ME (by volume), with a molar ratio CA:Ni of 6, and 70 mg/mL PVP. The phase-purity of the NiO fibers were confirmed using X-ray diffraction (XRD). The results of the experiments in which the polymer concentration was varied were in good accordance with a model reported by Shenoy et al., which was used to calculate the optimal polymer concentration for electrospinning [30]. Good results for NiO have also been obtained with acetylacetone or *N*,*N*-dimethyl formamide as solvents and/or complexing agents [31,32].

The ceramic microfibers after collection and thermal annealing are shown in Figure 4E. The conductivity and viscosity of the nickel solution were significantly higher than those of the 3YSZ solution (Table 1). As modeled by Thompson et al. [33], the jet’s momentum can be considered as a balance of electrical force counteracted by viscoelastic and surface tension forces. Feng reported a force balance of the jet as expressed by Equation 1 [23]:

−*p* + τ = *t*^e^ – γ/*R*, (1)

where *p* is the pressure drop related to the setup’s pump, τ the viscous stress, *t*^e^ the tension caused by the electrical field, γ the surface tension of the solution and *R* the jet radius. The electric force is governed by electrostatic charges in the solution, which can be correlated to the solution conductivity. The viscoelastic forces (τ) are correlated to the solution viscosity.

We observed comparable values for surface tension and a similar ratio between the viscosity and conductivity for both the nickel oxide precursor solution and the 3YSZ solution; looking at Equation (1), both solutions, therefore, experience an almost equal force contribution from surface tension and maintain a similar balance between viscosity- and conductivity-associated forces. The comparable electrospinning performance of these two solutions further corroborates the theory described by Equation (1) that a stable cone and jet are the result of a balance between viscoelastic, surface tension and electric forces [11,12,23,33], irrespective of their absolute magnitudes.

### 2.2. Process Parameters

**Figure 5:**
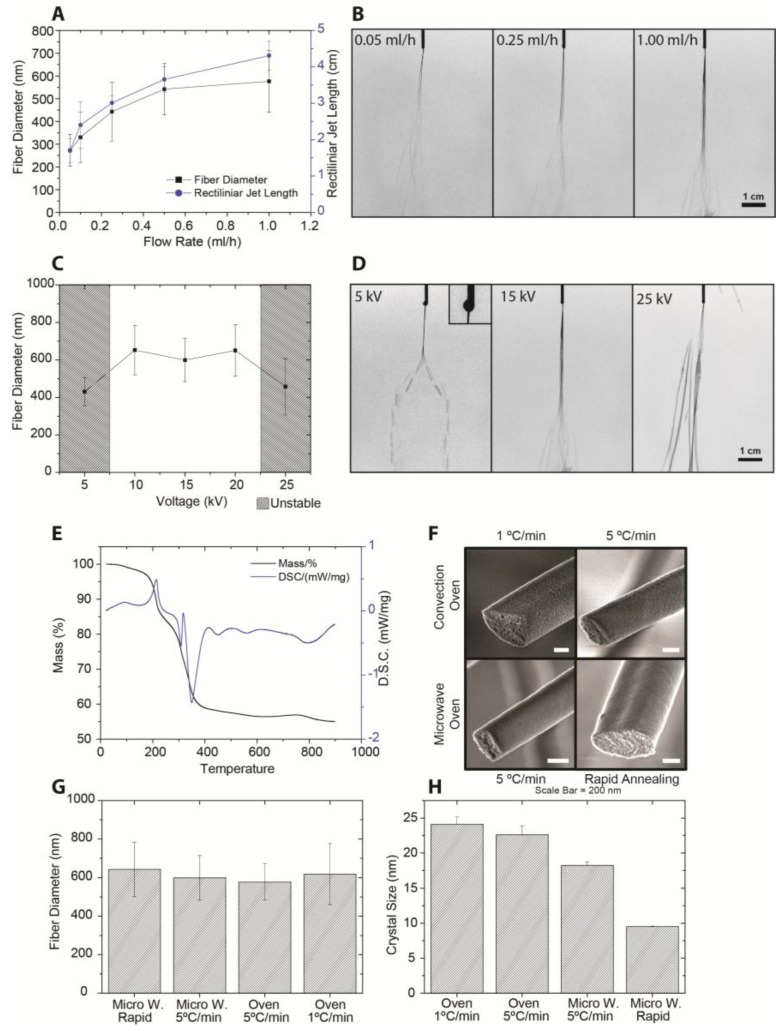
(**A**) Influence of flow rate on electrospinning process. Flow rate 0.05–1 mL/h. Fiber diameter (black squares) and rectilinear jet length (blue circles) at different flow rates are shown; (**B**) rectilinear jet length at flow rates of 0.05–1 mL/h; (**C**) influence of potential between spinneret and collector plate. Fiber diameter at voltages between 5 and 25 kV are shown; (**D**) electrospinning jet at voltages of 5–25 kV; (**E**) thermogravimetic analysis and differential scanning calorimetry (5 °C/min in air); (**F**) surface morphology of ceramic fibers after different annealing procedures; (**G**) fiber diameters after different annealing procedures; and (**H**) crystallite size of ceramic fibers after different annealing procedures.

It has been reported that a decrease of flow rate results in smaller polymeric fiber diameters [19]. Conversely, an increased flow rate has been thought to reduce the charge density in the jet [10,34], thus stabilizing the rectilinear jet region [10,11]. A longer stable jet implies a short whipping region and, thus, less fiber thinning [11], resulting in thicker fibers. We, therefore, investigated the influence of the flow rate on the rectilinear jet length and compared it with the final ceramic nanofiber diameter after collection. Indeed we observed that the rectilinear jet was longer when the flow rate was higher (Figure 5A,B), and the fiber diameter and rectilinear jet length followed the same trend.

The effect of the electric field strength on fiber diameter and jet length is presented in Figure 5C. The potential difference between spinneret and collector plate was varied between 5 and 25 kV, i.e., field strengths between 250 and 1250 V/cm, see Figure 5D. At 250 V/cm the solution was not fully electrified, and droplet formation was dominant. Between 500 and 1000 V/cm the solution was fully electrified and a stable jet was observed. At 1250 V/cm the solution was over-electrified, which resulted in an unstable jet that eventually sprayed (Figure 5D). The fiber diameter did not change within the voltage range where a stable jet was found (600 ± 100 nm). At field strengths < 500 V/cm, the fiber diameter was 430 ± 75 nm. We attribute the smaller diameter to the lower speed of the jet at a constant evaporation rate, which results in a shorter rectilinear jet and, thus, to a longer whipping region. At a 1250 V/cm field strength the fiber diameter was also reduced to 450 ± 150 nm. This can be understood by considering that, at very high voltages, the field strength stretches the jet and makes it whip even more, reducing the fiber diameter [18]. This phenomenon was confirmed by the observation of a highly unstable jet with a very short rectilinear jet length.

A convection oven and a microwave oven were employed to thermally treat YSZ nanofibers. Phase analysis using XRD confirmed the formation of phase-pure YSZ. The total mass loss was about 45% according to thermogravimetric analysis (Figure 5E). However, the surface morphology varied considerably with the mode of heating and the heating rate (Figure 5F), while the fiber diameter did not vary significantly (Figure 5G). Rougher surfaces were observed for samples annealed in a convection oven, which can be explained in terms of the heating mechanism [35,36,37]. Microwave heating has been reported to produce denser and smoother ceramic thin films than when prepared in a convection oven [36,37,38,39,40,41,42]. The precise mechanism of sintering using microwave radiation is not well understood [36,43], but the smoothness of the fibers is attributed to sudden shrinkage and densification, decreased step-bunching mechanism, and/or enhanced oxygen mobility resulting from the effect of microwave radiation on the polar solvents [36,40,41].

The sample heat-treated by rapid thermal annealing showed the smallest crystallite size, 9.5 ± 0.1 nm, whereas the sample with a slow heating rate of 1 °C/min had the largest size, 24 ± 1.0 nm. See Figure 5H. The differences can be attributed to the effective annealing time. At lower heating/cooling rates, a sample will remain longer at a temperature where grain growth can occur. However, the difference in grain size between two of the samples heated and cooled at 5 °C/min can only be attributed to differences in heating mechanism. Xie et al. reported smaller and more uniform grain sizes when zirconia was sintered in a microwave oven [37], and the nanofibers presented here may have undergone a similar process.

### 2.3. Hollow Fibers

**Figure 6:**
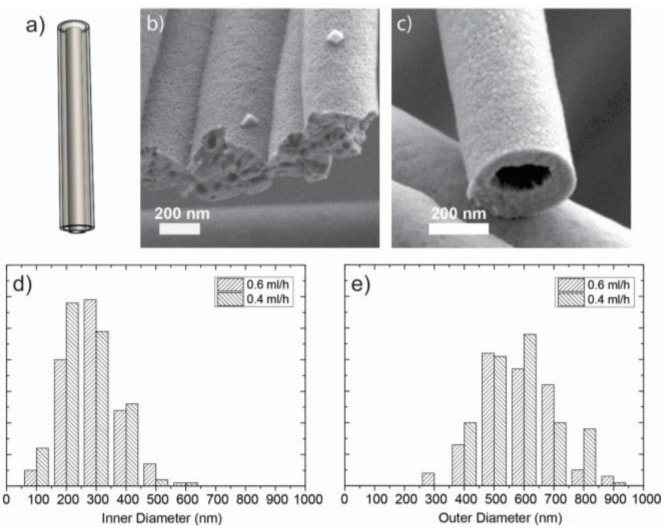
(**a**) Coaxial spinneret; (**b**) coaxial spinning of porous fibers. Inner flow rate is 0.2 mL/h PolyActive solution; Outer flow rate is 1 mL/h of 3YSZ precursor solution; no hollow fibers are formed; (**c**) hollow fiber made by coaxial electrospinning. Inner flow rate is 0.6 mL/h of the PolyActive solution; the outer flow rate rete is 1 mL/h of 3YSZ solution; (**d**) frequency distribution of the inner hollow fiber diameter at inner flow rates of 0.4 and 0.6 mL/h of the PolyActive solution. The outer flow rate is 1 mL/h of 3YSZ precursor solution; and (**e**) frequency distribution of the outer hollow fiber diameter under the same conditions. The fibers shown were thermally treated at 850 °C.

Ceramic hollow fibers were made using a spinneret with two concentric needles (Figure 6a). The 3YSZ precursor solution was pumped through the outer needle and an immiscible polymer solution poly(ethylene oxide terephthalate)/poly(butylene terephthalate (PEOT/PBT) through the inner needle. The outer flow rate was kept constant at 1 mL/h and the inner flow rate was varied from 0.2 mL/h to 1 mL/h. At flow rates below 0.4 mL/h hollow fibers could not be made as the inner polymer content was not enough to maintain a hollow fiber geometry, see Figure 6b. Dense fibers with isolated porosity formed. Ceramic hollow fibers were only formed at inner flow rates between 0.4 and 0.6 mL/h (Figure 6c). In this rather narrow regime the inner flow rate did not have an influence on the final diameter within experimental error. The annealed fibers had outer diameters of 530 ± 128 nm and inner diameters of 230 ± 93 nm (Figure 6d,e). Above 0.6 mL/h, the two immiscible solutions formed an emulsion and the jet became unstable. Spinning of hollow NiO nanofibers was not successful, which may be attributed to the relatively high conductivity of the salt solution.

### 2.4. Nanofiber Alignment

**Figure 7:**
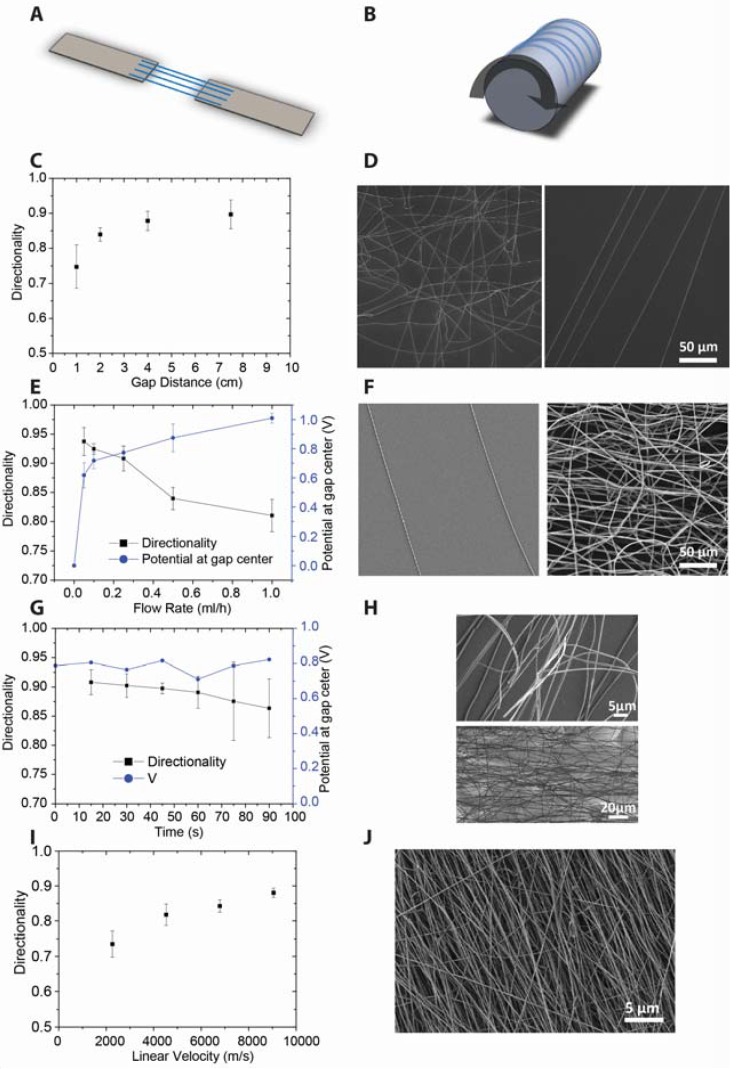
(**A**) Electrically-driven alignment: two ground electrodes with an insulating gap; (**B**) mechanically-driven alignment: rotating mandrel as the ground electrode. (**C**,**D**) Influence of gap distance on the degree of alignment of thermally-annealed fibers after 15 s of deposition. The flow rate is 0.25 mL/h; (**C**) degree of alignment versus gap distance; (**D**) SEM pictures of the samples spun with gap distances of 1.0 and 7.5 cm. (**E**,**F**) Influence of the flow rate of the 3YSZ precursor solution on the degree of alignment of fibers after 15 s of deposition. Gap distance is 2.0 cm; (**E**) degree of alignment and voltage versus ground at the gap center versus flow rate; (**F**) SEM pictures of the samples spun with flow rates of 0.05 mL/h and 1 mL/h. (**G**,**H**). Influence of deposition time on directionality of a sample spun at 0.25 mL/h of the 3YSZ precursor solution and a gap of 2.0 cm; (**G**) degree of alignment and voltage at the gap center versus time; (**H**) SEM image of a samples spun for 90 s. (**I**,**J**) Mechanical alignment of nanofibers with a rotating mandrel; (**I**) degree of alignment vs linear speed of the mandrel; (**J**) Sample spun for 30 min. All samples were spun at a flow rate of 1 mL/h of 3YSZ solution and thermally treated.

Experiments were carried out to align the as-synthesized nanofibers by electric field-driven alignment (Figure 7A) and by mechanically-driven alignment (Figure 7B). We recently demonstrated the use of array-like structures to fabricate a UV sensor and a field effect transistor (FET) device [44]. In field-driven alignment, two connected ground electrodes with a gap in between them is employed. The gap distance and flow rate were varied and the influence on the degree of alignment was investigated. Earlier studies on polymers showed an influence of gap distance on the degree of alignment of the fibers [13,45]. Simulations indicated that the lateral force by the electric field increases with gap distance, which favors alignment. The results of an experiment using YSZ precursor in which the flow rate was 0.5 mL/h and the gap distance was varied from 1.0 to 7.5 cm is shown in Figure 7C,D. The degree of alignment was lower at shorter gap distances than at larger gap distances, in agreement with previously reported data [13,45].

The influence of flow rate was studied by keeping the gap distance constant at 2.0 cm while varying the flow rate from 0.05 mL/h to 1 mL/h. The results are presented in Figure 7E,F. At very low flow rates, e.g., 0.05 mL/h, the alignment was nearly perfect, but the packing density of wires was low (Figure 7F). The degree of alignment decreased with the increasing flow rate. It is known that the electrical potential at the gap center plays a crucial role in the alignment process [13,45]. A near-zero potential at the gap center favors the lateral electrostatic forces that drive the fiber alignment process. In our experiments we observed that the electrical potential at the gap center increased with the flow rate, thus obstructing fiber alignment. Under steady-state conditions, there should be a balance between charges arriving from the jet and charges flowing to the electrode. The main cause of charge buildup in our experiments is probably the low conductivity of the hybrid fibers, preventing fast discharge to the electrodes.

Nanofiber alignment was also limited to short deposition times [7,15]. We observed a decrease of alignment in the course of time (Figure 7F,G). This probably occurred upon formation of thicker layers of fibers, where the bottom layer is thought to prevent new fibers from depositing on the electrodes, and hinder their discharge, so that the jet becomes unstable and starts to whip, leading to loss of alignment. An example is shown in Figure 7G.

Mechanical alignment presents an alternative method to align nanofibers (Figure 7B). We utilized a rotating mandrel as grounded electrode and a flow rate of 1 mL/h. The rotating speed of the mandrel was varied and the influence on alignment was quantified. The degree of alignment vs. linear velocity of the mandrel is plotted in Figure 7H. It can be seen that the alignment is better at higher speeds. This method allowed higher flow rates and longer deposition times to form thicker layers without influencing the alignment process negatively. Figure 7I shows a sample spun for 30 min at 1 mL/h. However, the alignment achieved with the gap method, which can easily reach 90–95%, is considerably better than the mechanical alignment, which is in the range of 70–90%.

## 3. Materials and Methods

### 3.1. Chemicals

Zirconium(IV) *n*-propoxide (Zr[(OC_3_H_7_)]_4_), 70 *w*/*w* % in propanol) and yttrium(III) acetate hexahydrate (Y(CH_3_COO)_3_·6H_2_O, purity 99.9%) were purchased from Alfa Aesar GmbH (Karlsruhe, Germany). 2-methoxyethanol (2-ME; 99.3%) and 1-propanol (99.9%) were acquired from Sigma-Aldrich (Zwijndrecht, Netherlands). Ethanol (99.8%) was purchased from Atlas and Assink Chemie bv (Enschede, Netherlands). Nickel(II) nitrate hexahydrate (Ni(NO_3_)_2_·6H_2_O) was acquired from Merck (Darmstadt, Germany), polyvinyl pyrrolidone (PVP, M_w_ 1,300,000) from Sigma-Aldrich (Zwijndrecht, Netherlands) and citric acid monohydrate (CA; 99.5%) from Alfa Aesar (Karlsruhe, Germany). All chemicals were used as received. A poly(ethylene oxide terephthalate)/poly(butylene terephthalate) (PEOT/PBT) copolymer was purchased from PolyVation BV. It consists of 45 wt % polyethylene oxide terephthalate and 55 wt % of polybutylene terephthalate. Chloroform (≥99%) was acquired from Sigma-Aldrich (Zwijndrecht, Netherlands) and 1,1,1,3,3,3-hexafluoro-2-propanol (HFIP) from Biosolve (Valkenswaard, Netherlands).

### 3.2. Electrospinning Solutions

Three percent yttria partially-stabilized zirconia (3YSZ) was taken as the model composition and was prepared using a metal alkoxide precursor. Solution preparation was done in a nitrogen atmosphere. Briefly, zirconium n-propoxide and yttrium acetate were dissolved in n-propanol at a molar ratio of 97:6, respectively. Then, 5–20 mg/mL of PVP was added to the solution and the solution was left stirring overnight to complete dissolution. Finally, it was placed into a syringe connected to the electrospinning setup.

The standard solution consisted of a 0.45–0.65 M solution of metal precursor and 10 mg/mL PVP. The polymer concentration was varied from 5 to 20 mg/mL PVP. We also investigated the formation of nickel oxide fibers from nickel salt solutions. A 3 M (stock) solution of Ni(NO_3_)_2_ in 2-ME was made and stirred overnight in air to allow complete dissolution. Additional isopropanol was added to bring the total volume fraction of 2-ME in the final solution to 0.4. Citric acid (CA) was added in a 6:1 molar ratio to nickel. 50 mg/mL PVP was added and the solution was diluted with n-propanol to bring the total concentration of Ni(NO_3_)_2_ to 0.21 M (taking the volume of PVP into account). Similar 0.21 M nickel solutions were made using ethanol, 2-ME and water. Solutions in 2-ME with CA in molar ratios CA:Ni of 2:1, 4:1 and 6:1 were also made. Finally, solutions in 2-ME containing 50, 70, or 100 mg/mL PVP were prepared.

We also performed coaxial electrospinning for the preparation of hollow ceramic fibers. Two immiscible solutions were spun using two concentric needles. The outer solution was the 3YSZ precursor solution, the inner solution was a polymeric solution consisting of 200 mg/mL PEOT/PBT in a 30:70 *v*/*v* % solution of chloroform:HFIP. After spinning, the inner sacrificial polymer was removed by thermal annealing, as further explained below.

### 3.3. Fabrication Parameters

An electrospinning setup equipped with a 0.8 mm spinneret was used. The standard parameters for 3YSZ were as follows: precursor flow rate, 1 mL/h; voltage 15 kV; spinneret to collector distance, 20 cm; relative humidity, 30%; temperature, 25 °C. We varied the flow rate from 0.05 to 1 mL/h and the voltage difference between the spinneret and collector plate from 5 kV to 25 kV. We kept the distance from the spinneret to the collector, humidity, and temperature constant to obtain and maintain a stable electrospinning process. In addition to the flat collector, we also used a grounded split electrode with an insulating gap between the electrode parts in order to obtain self-aligned fibers. The insulating gap varied from 2.0 to 7.5 cm width and the fibers were deposited on a silicon substrate. Instead of a split electrode we also used a rotating mandrel with a radius of 3 cm and a speed of 1000–4000 rpm to collect and orient the fibers. The annealing process was carried out in a convection oven at 850 °C for 2 h using heating and cooling rates of 5 °C/min. Samples were also annealed in a convection oven at 1 °C/min, or in a microwave oven at 5 °C/min, or by rapid thermal annealing. The rapid thermal annealing process involves placing the sample in a preheated microwave oven up to 850 °C.

The electrospinning parameters for the nickel oxide precursor solution were as follows: flow rate, 0.6 mL/h; voltage difference, 15 kV; spinneret to collector distance, 15 cm; relative humidity, 30%; temperature, 25 °C. Coaxial spinning was performed using a spinneret from SpinBow. The inner needle had a diameter of 0.3 mm and the outer needle had a diameter of 0.8 mm.

### 3.4. Characterization

Static viscosity measurements were performed using an AMVn microviscometer (Anton Paar, Graz, Austria) at 25 °C, using a 3 mm capillary with matching 2.5 mm steel ball (1.4034 g/cm^3^) under an 80° angle. Dynamic viscosity measurements, performed on an Anton Paar Physica MCR 501 (Anton Paar, Graz, Austria), were done to prove the thinning behavior of the solutions at high shear rates. Conductivity measurements were performed using a home-made two-point probe, consisting of two parallel Pt wires inserted perpendicularly to a gap in an alumina tube. The wires were fixed with a Torr Seal^®^ epoxy resin to prevent the solution to penetrate into the tube and ensure contact with the parallel region of the wires only. The probe was connected to an Autolab PGSTAT128N potentiostat/galvanostat (Metrohm Autolab, Utrecht, Netherlands). The data were collected using NOVA 1.9.16 software. A frequency sweep measurement was done between 10 kHz and 1 Hz with an amplitude of 10 mV. The solution was kept at 25 °C. The conductivity was calibrated with standard KCl solutions with known conductivities [46,47,48].

Scanning electron microscope (SEM) pictures to investigate the microstructure were taken with a Merlin Scanning Electron Microscope (Carl Zeiss, Jena, Germany). Pictures of the electrospinning jet were taking utilizing a Nikon D500 camera (ISO 5000 and shutter speed of 1/200 s) (Nikon Nederland, Amsterdam, Netherlands) equipped with a Carl Zeiss 100 mm lens.

The surface tension of the precursor solutions was measured with the pendent droplet method using a contact angle system OCA (DataPhysics Instruments GmbH, Filderstadt, Germany). The results were analyzed with SCA20 software (DataPhysics Instruments GmbH, Filderstadt, Germany). We quantified the alignment of the fibers with Fiji ImageJ software [49]. We used a Nikon Eclipse ME600 optical microscope (Nikon Nederland, Amsterdam, Netherlands) and scanning electron microscopy (SEM) for the electrically- and mechanically-aligned fibers. The directionality tool of Fiji ImageJ provides a histogram with a preferred orientation and dispersion (standard deviation) over 180°. We defined the degree of alignment as follows:

Degree of alignment = 1 − dispersion/90
(2)

We measured the charge buildup of the electrically-driven alignment by monitoring the voltage between one of the ground electrodes and a platinum electrode at the center of the gap using a Keithley 197 Voltmeter. Phase analysis was done using powder X-ray diffraction (XRD) with a Bruker D2 Phaser (Cu Kα radiation λ = 0.15405 nm) (Bruker Nederland bv, Leiderdorp, Netherlands). Thermogravimetric analysis and differential scanning calorimetry were performed in a Netzsch STA 449 F3 Jupiter Thermal analyzer (Netzsch, Selb, Germany). The sample was heated with a rate of 5 °C/min in air to 900 °C.

## 4. Conclusions

This study demonstrated that electrospinning allows the fabrication of dense and hollow ceramic fibers with controlled dimensions. The fiber diameter can be varied from hundreds of nanometers to more than a micrometer. The upper and lower limits to the fiber diameter are governed by the properties of the precursor solution used in the spinning process.

Electrospinning also allows a considerable degree of control in the fabrication of arrays of aligned fibers and hollow fibers. A limitation of the technique appears to be that the inner and outer diameters of the hollow fiber cannot be modified independently. With respect to field-driven fiber alignment, there is a limit to the number of fibers that can be aligned, while the degree of alignment is intrinsically poorer when it is mechanically driven.

Nevertheless, electrospinning has been proven to be a useful technique to produce ceramic nanofibers, with the possibility to control their microstructure and properties. It offers a unique combination of control over fiber structure at relatively high production rates, which makes it a promising tool to produce dedicated nanofiber materials with unique properties.

## Figures and Tables

**Table 1:** Solution properties of three percent yttria partially-stabilized zirconia (3YSZ) and NiO precursor solutions. 3YSZ solution has a precursor concentration of 0.65 M and PVP concentration of 10 mg/mL. NiO precursor solution has a precursor concentration of 0.21 M in 2-ME with 70 mg/mL of PVP and citric acid in 6:1 CA:Ni molar ratio.

	3YSZ	NiO
η (mPa·s)	46 ± 0.15	325 ± 2
σ (μS/cm)	58 ± 0.07	397 ± 5
γ (mN/m)	9.45 ± 0.07	9.58 ± 0.05
